# Characterization of darter (*Etheostoma* spp.) interspecific energetic responses to acute temperature elevations

**DOI:** 10.1093/conphys/coaf027

**Published:** 2025-04-15

**Authors:** Allison V Weber, Paul M Craig

**Affiliations:** Department of Biology, University of Waterloo, 200 University Ave West, Waterloo, ON N2L 3G1, Canada; Department of Biology, University of Waterloo, 200 University Ave West, Waterloo, ON N2L 3G1, Canada

**Keywords:** Aerobic scope, climate change, Ct_max_enzymatic activity, heatwaves, metabolism, small-bodied fishes **Abbreviations:** FTD, fantail darter; RBD, rainbow darter; JD, Johnny darter; PK, pyruvate kinase; LDH, lactate dehydrogenase; MDH, malate dehydrogenase; CS, citrate synthase; COX, cytochrome c oxidase; AS, aerobic scope; CT_max_, critical thermal maximum; LOE, loss of equilibrium

## Abstract

Understanding metabolic responses to temperature elevations is critical for determining how fish populations will be impacted by the increased occurrence of extreme heat events. Here, we characterized the thermal tolerance limits and metabolic functions of three closely related darter species native to the Grand River of Southern Ontario: Fantail darter *(Etheostoma flabellar*e; FTD), Rainbow darter (*Etheostoma caeruleum;* RBD) and Johnny darter (*Etheostoma nigrum;* JD). Brain and heart activity of enzymes associated with cellular respiration were analysed for each species at 15°C baseline and following a Critical Thermal Maximum (CT_max_) test. Additionally, aerobic scope (AS) was determined for each species while exposed to four heat ramps designed to mimic previously recorded heatwaves. CT_max_ significantly differed between species with FTD displaying the highest at 33.3°C, JD second at 31.8°C and RBD the lowest at 30.7°C. In darters not exposed to heat stress, FTD possessed higher brain enzymatic activity rates, specifically in pyruvate kinase (PK), citrate synthase (CS) and malate dehydrogenase (MDH). These patterns shifted slightly after exposure to CT_max_, with JD displaying a substantial elevation in PK, lactate dehydrogenase, CS and MDH activity, suggesting they had greater enzymatic capacity at temperature extremes. Within heart tissue, we observed no interspecific differences at baseline temperatures; however, RBD had lower enzyme activity than FTD or JD in all enzymes but cytochrome c oxidase following CT_max_. Metabolically, FTD exhibited the highest AS following exposure to 10 and 15°C temperature elevations. Our findings demonstrate that FTD may be the best equipped to respond to temperature-induced increases in metabolic demand due to their elevated baseline enzymatic activity and broader AS. These insights may contribute to future darter conservation efforts by informing predictions on species population shifts, particularly in the context of climate change.

## Introduction

Freshwater environments are expected to experience elevations in temperature variability as the occurrence and severity of extreme heatwaves, and daily temperature maximums continue to rise with the progression of global climate change (Geerts, 2003; Meehl and Tebaldi, 2004; Frölicher *et al*., 2018; Stillman, 2019). Environmental disturbances like loss of shade cover and warm water effluent from urban heat islands and thermal power plants introduce further temperature variation to these aquatic ecosystems (Lynch *et al*., 1984; Somers *et al*., 2013; Coulter *et al*., 2014; Kalny *et al*., 2017; Mohajerani *et al*., 2017), These culminate in adverse impacts on aquatic species, particularly those that reside in shallow-water habitats that lack temperature buffering capacity (Morash *et al*., 2021). Ectothermic organisms such as fish are especially vulnerable to temperature changes as they possess no internal mechanisms of maintaining body temperature, making their physiological conditions directly regulated by environmental temperatures (Fry, 1947; Schulte *et al*., 2011). Among fishes, temperature is known as the ‘master abiotic factor’ due to its integral role in physiological functioning, particularly through its influence on biochemical reaction rates involved in metabolism (Fry, 1947; Schulte *et al*., 2011; Schulte, 2015). Consequently, thermal composition of habitats directly impact metabolic functioning. This in turn determines, and potentially limits, the metabolic capacity of fishes for both physical and physiological activity, thereby affecting their ability to respond to different environmental conditions, changes or challenges (Brown *et al*., 2004, 2012). These limitations thus present larger implications on species fitness, population shifts and geographical distributions (Somero, 2005; Sunday *et al*., 2012).

In light of the temperature dependency of fish, analysis of metabolic function under temperature elevations is essential for predicting how species will respond to extreme heat events and determining the future implications of climate change on fish populations. In ectothermic fish, metabolic rate can be broken into three different types, depending on the level of energy requirement. When energy demands are minimal, directed mainly towards sustaining homeostasis, and the fish is resting in a post-absorptive and non-reproductive state, the fish is said to be at standard metabolic rate (SMR; Chabot *et al*., 2016). For situations of spontaneous, voluntary or ‘routine’ activity, routine metabolic rate (RMR) is reached, which tends to be more environmentally relevant than SMR considering wild species are rarely at basal and quiescent levels (Norin and Speers-Roesch, 2021). At the uppermost limits of energetic demand, usually following exhaustive exercise, maximum metabolic rate (MMR) occurs (Norin and Clark, 2016). Comparing the respective values of each metabolic rate can elucidate invaluable information on how fish interact with their environment. Often, this is assessed via aerobic scope (AS), the difference between MMR and SMR, which depicts how much metabolic capacity is available beyond the requirements needed for basic physiological maintenance (Pörtner, 2001; Clark *et al*., 2013). AS therefore provides a metric for determining the metabolic capacity left over for responding to environmental challenges, and has been used extensively in fish physiology to address a range of ecologically relevant questions (Pörtner and Knust, 2007; Farrell *et al*., 2008; Schulte, 2015). These relationships are temperature-dependent and grounded in the rationale that an optimum temperature range exists at which AS peaks, wherein performance and physiological functioning are optimized (T_optAS_; Pörtner and Knust, 2007). At temperatures lower or higher than T_optAS_, AS drops off, producing an overall bell-shaped curve (Schulte *et al*., 2011). Prior work has shown high AS in sockeye salmon (*Oncorhynchus nerka*) to be associated with spawning migration success, while failed migrations were attributed to a collapse in individual AS (Farrell *et al*., 2008; Eliason *et al*., 2011). Similarly, high T_optAS_ observed in pink salmon (*Oncorhynchus gorbuscha*) was theorized as a reason for their recent range expansion as northern water temperatures warm (Clark *et al*., 2011). Together these findings highlight the insight AS provides into determining which fishes may be most capable of handling energetic stress or temperature increases from climate change.

When environmental temperatures surpass the organism’s T_optAS_ and approach tolerance limits, a range of sublethal, physiological effects occur, with any deviations past temperature thresholds having lethal consequences (Ficke *et al*., 2007). The upper temperature at which physiological functioning is no longer possible, Critical Thermal Maximum (CT_max_), is understood as the thermal tolerance limit of the respective organism, and commonly considered the point at which the organism would die in the wild (Lutterschmidt and Hutchison, 1997; Ern *et al*., 2023). The physiological mechanisms behind thermal tolerance are still debated; however, the failure of brain and heart tissue specifically, are believed to be implicated in determining upper temperature limits (Ern *et al*., 2023). Previous studies have reported decline in heart function when nearing thermal tolerance thresholds, and determined it often is one of the first organs to fail at temperature extremes (Becker and Genoway, 1979; Farrell, 2002; Mendonça and Gamperl, 2010; Ern *et al*., 2023). Similarly, recent developments from Andreassen *et al*. (2022) posited that lack of oxygen delivery to the brain, which has high energy requirements but low energy reserves, may be responsible for determining thermal tolerance limits, explaining the locomotor dysfunction known as loss of equilibrium (LOE) that often occurs at CT_max_ in fish (Becker and Genoway, 1979; Soengas and Aldegunde, 2002; Andreassen *et al*., 2022).

Like AS, CT_max_ has numerous applications in drawing broader conclusions on the capabilities of fishes at handling climate change, and although experimentally conducted on an acute temporal scale, CT_max_ values have demonstrated correlations to longer term heat exposure tolerances, suggesting applicability of CT_max_ to heatwaves in the wild (Åsheim *et al*., 2020). However, these extrapolations of CT_max_ to the broader environment are still somewhat debated as recent work on Atlantic salmon (*Salmo salar*) observed no relationship between CT_max_ and tolerance to more chronic incremental temperature increases (Bartlett *et al*., 2022). Yet, in sockeye salmon, higher CT_max_ was reported in populations with difficult migrations, demonstrating its potential ability to provide insight into species’ capacity to handle energetic challenges in the wild.

While AS and thermal tolerance analyses have been employed in numerous salmonid species to determine their relative abilities of responding to environmental temperature changes or challenges (Farrell *et al*., 2008; Clark *et al*., 2011; Eliason *et al*., 2011; Chen *et al*., 2013), fewer studies have been done on small, freshwater species that make up a large percentage of the biomass in streams and rivers and play a key role in ecosystem food webs (Small, 1975; Kuehne and Barbour, 1983). As such, a large knowledge gap exists in our understanding how these small and often overlooked species will be impacted by the predicted environmental shifts associated with climate change, particularly their metabolic responses to elevated temperatures.

Responses of fishes to climate change will vary across species based on their respective sensitivities and resiliencies to environmental perturbations, likely determined by behavioural responses, genetic diversity of populations and physiological tolerances (Williams *et al*., 2008), of which is known to be influenced by species-specific differences in prior acclimatization, adaptation and evolutionary history (Comte and Olden, 2017). *Etheostomatinae*, commonly referred to as darters, are small benthic perches endemic to freshwater rivers and streams across North America (Kuehne and Barbour, 1983). Although highly common, a relatively small amount of physiological research has been done on darters and even less on determining how climate change will affect these species. While previous studies have examined impacts of temperature increases on darters in terms of habitat elevation (Troia and Giam, 2019,), or compared darter AS values relative to invasive species (White *et al*., 2017), virtually no studies have examined how darters may respond metabolically to acute temperature spikes associated with climate change, aside from recent work done by Borowiec *et al*. (2024), which reported Rainbow darters (*E. caeruleum*) having rapid metabolic recovery to acute temperature stress and exhaustive exercise (Borowiec *et al*., 2024). Consequently, little is known on the metabolic capacity of darters to handle temperature increases, presenting broader questions on which *Etheostoma* species may be most capable of tolerating climate change, and what this might mean for North American riverine ecosystems as a whole.

To fill these gaps, we characterized the thermal tolerance and respective metabolic responses of three closely related species of darter: Fantail *(E. flabellare;* FTD), Rainbow (*E. caeruleum:* RBD) and Johnny darter (*E. nigrum;* JD). Although often co-existing and residing within similar overall temperature ranges of ~4–20°C, each species prefers a distinct microhabitat within a given section of the river: JD favouring pools, FTD occupying shallow riffles and RBD inhabiting deep, fast-flowing runs. These habitat differences suggest they may experience different temperature profiles or variability. Here, our objectives were to determine the CT_max_ between species, and characterize their differences in energetic enzymatic activity both within and between species at river temperatures and at their thermal tolerance limits. We also sought to analyse darter metabolic responses, specifically AS, following exposure to environmentally relevant temperature increases. We predicted that, given their microhabitat temperature makeup, darters would exhibit different thermal tolerances and metabolic capabilities when handling acute temperature elevations.

## Materials and Methods

### Fish collection and husbandry

Two cohorts of fish were collected, one in October 2022 for CT_max_ and enzyme analyses (*n* = 40 per species), and another in October 2023 for assessment of metabolic rate (*n* = 20 per species). For both cohorts, adult RBD, JD and FTD of mixed sex were collected from the Grand River at West Montrose (43°35′08.7”N 80°28′53.4”W) via backpack electrofishing (Smith-Root, 150 V, 30 Hz, 15 ms). Darters were brought back to the Waterloo Aquatic Threats in Environmental Research (WATER) facility at the University of Waterloo and housed in a 385-l Z-HAB rack system (Pentair Aquatic Eco-Systems Inc., Apopka, Florida, USA) with ~12 fish, separated by species, per 10-l acrylic tank. Tanks were supplied with dechlorinated, reverse osmosis, softened city water, and maintained at a temperature of 15 ± 0.5°C and pH of 8.0 to match conditions recorded from the catch site. For all studies, fish were acclimated for 2 weeks prior to experimentation, during which they were fed frozen bloodworms to satiety daily, and kept under 12:12 h light:dark cycles, with 0.5-h ramp transitions. Considering darters are benthic species, housing structures were placed in each tank for enrichment. All experiments performed were in accordance with the Canadian Council of Animal Care guidelines as reviewed by the University of Waterloo Animal Care Committee (AUP #44638).

### Critical thermal maximum

Thermal tolerance limits were determined via CT_max_ thermal test, with all fish fasted 24 h prior to the CT_max_ procedure. Five trials were conducted per species, each trial consisting of four fish total with two fish of a single species placed in each of two, 16.5 × 12.5 × 13 cm mesh breeding boxes, resulting in *n* = 20 per species. Breeding boxes were fitted side by side in a 30-l glass aquarium water bath and left to acclimate for 15 min at 15°C. Temperature of the water bath was then increased incrementally by 0.33°C per minute through a Julabo portable immersion circulator (Becker and Genoway, 1979; Seelbach, Baden-Württemberg, Germany). Significant mixing of warmed water was performed by a water pump on the Julabo circulator, with warm water diffusively entering the mesh box. Fish were visually monitored, and occasionally prodded with a probe to ensure LOE, the point at which the fish became unable to maintain its position in the water column, going belly up (Becker and Genoway, 1979). Water temperature, monitored by the Julabo circulator, was recorded with the occurrence of LOE. Afterward, the fish were removed from the mesh box and immediately euthanized using buffered 0.5 g/l ethyl 3-aminobenzoatemethanesulfonate (MS-222; Sigma-Aldrich). Brain and heart tissue were removed, flash frozen on dry ice, and stored at −80°C until further use. No mortalities occurred during any of the CT_max_ trials.

### Enzyme assays

Enzyme activity of brain and heart tissue was determined via enzyme assay for all darter species for both those sampled at their respective thermal tolerance limits and at a baseline temperature of 15°C. Baseline fish were sampled after completion of the 2-week 15°C lab acclimation, with CT_max_ trials conducted in the following 2 days. Key metabolic enzymes were examined: pyruvate kinase (PK) and lactate dehydrogenase (LDH), involved in glycolytic reactions; citrate synthase (CS) and malate dehydrogenase (MDH), involved in the citric acid cycle; and cytochrome c oxidase (COX), involved in the electron transport chain.

Frozen brain and heart tissue were homogenized on ice in 20 ml of buffer per 1 mg of tissue (20 mM Hepes, 1 mM EDTA, 0.1% Triton X-100, pH 7.4, 1 anti-protease pill/10 ml of buffer) via OMNI TH handheld tissue homogenizer (Kennesaw, Georgia, USA). Homogenates were then centrifuged (12 000 g, 10 min, 4°C) and supernatants removed for use in enzyme activity assays. All assays were performed in 96-well microplates and measured for 10 min at 25°C using a Molecular Devices Spectramax 190 spectrophotometer (San Jose, California, USA) in tandem with the SoftMax Pro 6.3 software. The assay temperature of 25°C was chosen for assays of both baseline and CT_max_ treatment groups due to physical limitations of the spectrophotometer at holding temperatures of 15°C and >30°C. Cytosolic enzymes, PK and LDH, were measured first after one freeze–thaw cycle; mitochondrial enzymes, CS, MDH and COX, were measured after two freeze–thaw cycles to allow for sufficient opening of mitochondria. All freeze–thaws were consistent across all samples within each enzyme. PK, LDH and MDH were all measured at 340 nm, CS at 412 nm and COX at 550 nm. Enzyme reaction buffers were made according to the protocols outlined in Mehdi *et al*. (2018) and Dawson *et al*. (2016). The remaining sample was used in Bicinchoninic acid assays to determine protein quantity of each sample and used to normalize enzyme activity to protein content. For all enzyme assays conducted, a sample size of *n* = 10 per group was used.

### Respirometry set-up

Darter metabolic responses to elevated temperature were characterized through measurement of oxygen consumption (ṀO_2_) via intermittent-flow respirometry. This method alternates between periods of active and inactive water flow through sealed holding chambers, with pump switching controlled by Loligo AutoResp v3 software (Vborg, Jutland, Denmark). During the activated water flow cycle, known as the flush, fresh water is pumped from the surrounding water through the chamber and expelled. The deactivated water flow period, known as the measurement cycle, pumps water in a closed loop from the chamber across a fibre-optic O_2_ sensor (PreSens, Regensburg, Germany), which measures the decline in oxygen within the sealed chamber at 1-s intervals. In between the flush and measurement cycles is a short wait period wherein the flush pumps are turned off and measurements are turned on, priming the closed loop for measurement without data collection. Here, our set-up consisted of eight, 150-ml cylindrical glass chambers immersed in a ~50-l insulated, plastic trough, with one chamber left empty to determine background respiration. Aquaria airline tubing was used in the flush and closed loops, with the volume of the closed loop tubing, 25 ml, accounted for in respiration calculations. O_2_ sensors were calibrated every 2 weeks via two-point method of exposure to 100% air saturation, and 0% air saturation, using a 20 g/l Na_2_SO_3_ solution, as per Borowiec *et al*. (2024). We employed a 3-min flush period, 30-s wait and 3-min measurement, which was sufficient time to allow chamber oxygen concentration levels to return to full saturation during the flush period.

### Respirometry procedure and calculations

**Figure 1:**
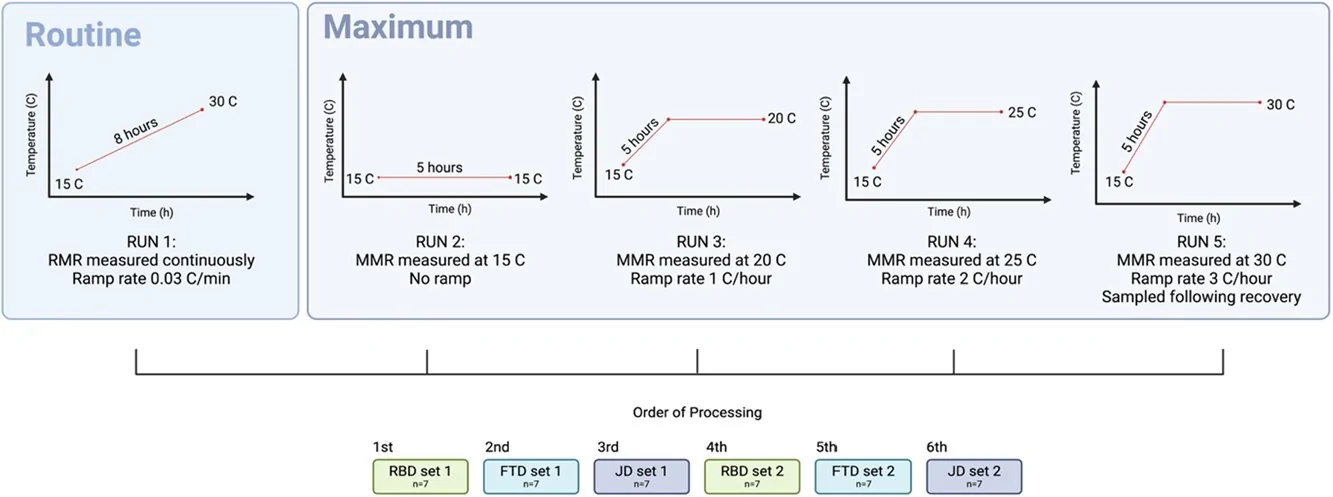
Respirometry experimental schematic. Intermittent-flow respirometry was utilized for determination of routine and maximum oxygen consumption (ṀO_2_) in JD (*n* = 14), RBD (*n* = 14) and FTD (*n* = 14) darters, following five different temperature ramps. All fish were placed into chambers at 15**°**C overnight prior to measurement. Each species set rested for 1 week following trial. Created in BioRender. Craig, P. (2025) https://BioRender.com/nrvyufl

Metabolic rates of 14 fish per species were determined while exposed to five different heating profiles, specifically, 15–30°C over 8 h (henceforth referred to as 15°C slow ramp), 15°C held over 5 h (0°C fast), 15–20°C over 5 h (5°C fast), 15–25°C over 5 h (10°C fast) and 15–30°C over 5 h (15°C fast). Each species was divided into two sets of seven fish that cycled through each of the temperature profiles, working through set 1 of each species first followed by set 2. For example, the order would proceed as follows: RBD set 1 (*n* = 7) exposed to the 15°C slow ramp, then FTD set 1 (*n* = 7), JD set 1 (*n* = 7), RBD set 2 (*n* = 7), FTD set 2 (*n* = 7) and lastly JD set 2 (*n* = 7), with each species set taking one full day per temperature profile (Fig. 1) This procedure allowed for each set to have 1 week of rest and feeding after each respirometry trial. As such, each temperature profile took roughly 1 week to complete, resulting in a total experimental duration of 5 weeks.

At 17:00, after fasting for 24 h, seven fish of one species were removed from their holding tanks, weighed and placed into chambers, with fish capable of viewing each other. Once in their respective chambers, a privacy shade was placed around the perimeter of the trough to eliminate external disruptions, and fish were left to acclimate overnight at 15°C. Without removing the privacy shade, temperature ramp profiles were started at 08:30 the following morning, when the 50-l water bath was heated via Julabo portable immersion circulator (Seelbach, Baden-Württemberg, Germany) specifically programmed for each thermal profile through the EasyTemp software. After completion of the fast, 5-h temperature ramps, water bath temperature was held at the upper limit of the ramp as fish were individually removed from their chambers for a 2-min air exposure to determine MMR. Air exposure was used in lieu of the typical chase protocol due to previous reports of darter inactivity in response to a chase stimulus (Roche *et al*., 2013; Hodgson *et al*., 2020). The remaining fish chambers not undergoing the air exposure were covered to minimize added stress associated with human interaction. Across all trials, the 50-l water bath was filtered and temperature monitored at both ends of the trough to ensure consistent temperature ramping in all areas of the water bath. Following completion of each trial, the trough was completely emptied and cleaned with fresh water, then refilled before placing the next round of fish in to acclimate overnight.

Raw oxygen consumption rates recorded by the Loligo software were converted to ṀO_2_ by multiplying by the chamber volume (minus the fish’s displacement) and dividing by fish mass to determine mass-specific ṀO_2_ per individual fish. Background respiration of the blank chamber of each trial was subtracted from all ṀO_2_ chamber values of that specific trial. The 15°C slow ramp was used for determination of RMR at temperature points along the ramp; 15, 20, 25 and 30°C, while the other four, fast trials, were used for determination of MMR via air exposure at the upper temperature limits of each trial; 15, 20, 25 and 30°C. Absolute AS was then calculated for each individual at each of the four temperature points, 15, 20, 25 and 30°C, by subtracting the single, lowest RMR rate out of three values for each given temperature from the MMR value of the same temperature, with MMR deemed the single rate of oxygen consumption measured immediately upon the fish’s return to the respirometry chamber following air exposure. We acknowledge that our calculation of AS (MMR – RMR) differs from the traditional definition (MMR – SMR), due to the inability to measure SMR along the temperature ramp from 15 to 30°C. However, we believe that using RMR in AS calculations may provide a more realistic reflection of actual aerobic capacity, considering fish are rarely at basal metabolic levels in the wild.

### Statistical analyses

All statistical analyses, unless stated otherwise, were performed by GraphPad Prism 8.1.2 using a significance level of α = 0.05 (GraphPad, San Diego). All data were tested for normality and lognormality, homogeneity of variances (via Barlett’s and Brown–Forsythe test) and normality of residuals. Datasets that failed to pass normality were log transformed to satisfy statistical assumptions of normality. An Analysis of Covariance (ANCOVA) was conducted in RStudio (Ver. 4.3.3) to assess for any impacts of body mass on CT_max_. Considering there was no effect of mass, a one-way Analysis of Variance (ANOVA) with a Tukey Honest Significant Difference (HSD) test was performed to determine significant differences in CT_max_ values between RBD, JD and FTD species. For brain tissue, all enzyme activity data were log transformed; heart enzymatic data were normally distributed except for COX, which was log transformed. Differences in enzyme activity between temperature treatment groups and species were assessed by a two-way ANOVA with Tukey HSD test. When a significant interaction between the CT_max_ treatment and species was reported, data were split individually by variable and re-analysed to assess for differences between species at each heat treatment, determined by one-way ANOVA with Tukey HSD test, or differences within species across heat treatments, determined via *t*-test.

**Figure 2:**
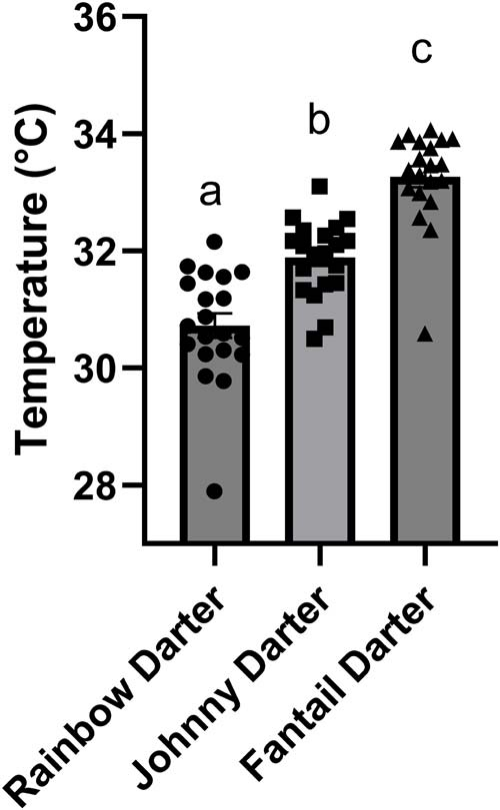
Rainbow, Johnny and Fantail darter Critical Thermal Maximum differences. Different letters denote significant differences between groups (*P* < 0.05; *n* = 20; 2 CT_max_ trials with *n* = 10 per trial) as determined via one-way ANOVA and Tukey HSD test. Dots represent individual fish. Bars represent mean ± standard error.

Body mass of individual fish used in the respirometry experiment was ln transformed and plotted against AS to determine linear model fit. All species were deemed linear with R^2^ values reported in Supplementary Table S1. AS data were then analysed via two-way ANCOVA to determine the effect of mass on oxygen consumption. Mass was a significant covariate (Supplementary Table S2), consequently, AS between species and 15, 20, 25 and 30°C was analysed by performing a linear mixed effects model that included mass as a covariate and accounted for the repeated measures nature of the data via fish ID as a random effect. A significant interaction between species and heat treatment was found. As a result, data were separated based on variable, and analysed again via linear mixed effects model, with mass still included as a covariate, and followed by a Tukey HSD test. Additionally, mass and Fulton’s condition factor (K), often used to provide insight on the overall health of a fish, calculated (Weight (g)/Length (cm)^3^)*100, were compared between species by one-way ANOVA with Tukey HSD test, to gain insight on the effect of mass on MO_2_ for each species. Traditionally used, mass-corrected ṀO_2_ (mg O^2^ kg^−1^ h^−1^) data, in which data points are divided relative to the mass of the fish, was also analysed via linear mixed effect model with Tukey HSD test, for methodological comparison with the data that directly included mass as a covariate. Respirometry data were assessed for species and body mass interaction via ANCOVA, to determine if any differences in mass scaling of AS existed between RBD, JD and FTD. All ANCOVAs were performed on RStudio**.**

## Results:

### Critical thermal maximum

Thermal tolerance limits (CT_max_) were significantly different between all three darter species (one-way ANOVA, F_2,57_ = 50.54, *P* < 0.0001) with FTD expressing the highest CT_max_ (33.3°C ± 0.79), followed by JD (31.9°C ± 0.64) and RBD (30.7 ± 0.94; Fig. 2). Mass had no impact on the CT_max_ of any species (Supplementary Table S3).

### Energetic enzyme activity

**Figure 3:**
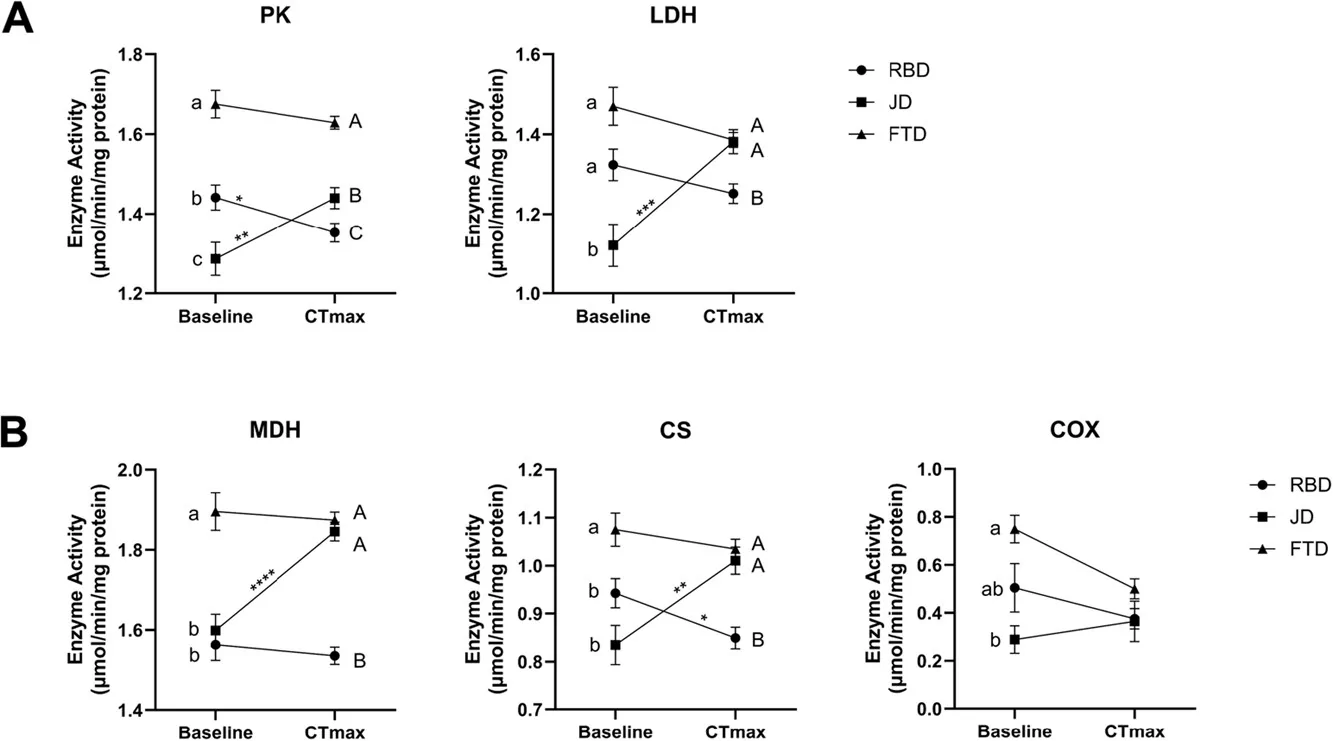
Normalized enzyme activity of RBD, JD and FTD brain tissue at temperature baseline (15°C) and at CT_max_ in cytosolic (A) and mitochondrial (B) enzymes. Interspecies differences in activity at baseline denoted by different lowercase letters, interspecies differences in activity at CT_max_ denoted by different uppercase letters. Intraspecies differences between Baseline and CT_max_ denoted by stars above corresponding lines (*P* ≤ 0.05 = *, *P* ≤ 0.01 = **, *P* ≤ 0.001 = ***, *P* ≤ 0.0001 = ****). All differences were determined by a two-way ANOVA with Tukey HSD test (*P* < 0.05; *n* = 10) presented as mean ± SEM. When interaction was significant, data were split by variable and reassessed via one-way ANOVA for interspecific differences, or *t*-test for intraspecific differences. All data were log transformed to achieve normality.

In brain tissue, all enzymes but COX expressed an interactive effect between species and heat treatment (Supplementary Table S4), thus only COX activity was analysed via two-way ANOVA with Tukey HSD test when the main effect variable, either species or heat treatment, was significant. Of the brain cytosolic enzymes examined within heat treatments via one-way ANOVA (Fig. 3A; Supplementary Tables S5 and S6), FTD appeared to have a baseline advantage, demonstrating 141% higher PK enzymatic activity (48.7 ± 11.7 μmol/min/mg protein) than JD (20.1 ± 5.5), 71% higher than RBD (28.3 ± 7.2) and greater LDH (31.3 ± 12.2) activity over JD (14.1 ± 5.0). At CT_max_ however, these relationships flipped, and RBD activity became the lowest for both cytosolic enzymes (PK: 22.8 ± 4.0, LDH: 18.1 ± 3.1), although FTD maintained elevated activity levels at temperature extremes (PK: 42.8 ± 5.2, LDH: 24.5 ± 3.4). JD often had increased activity at CT_max_ (PK: 28.0 ± 5.5, LDH: 24.6 ± 5.4) compared to baseline (PK: 20.1 ± 5.5, LDH: 14.1 ± 5.0), while RBD activity tended to be lower at CT_max_ (PK: 22.8 ± 4.0) relative to its baseline counterparts (PK: 28.3 ± 7.2), seen in PK (Supplementary Table S7). FTD demonstrated no intraspecific differences across heat treatment (Supplementary Table S7). Brain mitochondrial enzymes when examined interspecifically had similar trends as the cytosolic fractions (Fig. 3B; Supplementary Tables S5 and S6). For MDH and CS, FTD had higher baseline activity (MDH: 83.6 ± 36.0, CS:12.2 ± 3.4) compared to JD (MDH: 41.0 ± 11.2, CS: 7.1 ± 2.0) and RBD (MDH: 37.9 ± 10.7, CS: 9.0 ± 2.0) and higher COX (6.1 ± 2.7) than JD (2.1 ± 2.7).

Although FTD still showed higher MDH (75.6 ± 11.0) and CS (10.9 ± 1.7) activity levels than RBD (MDH: 34.7 ± 5.9, CS: 7.2 ± 1.2), at CT_max_ the difference over JD seen at baseline was lost, due to a JD-specific 72% increase in MDH (71.0 ± 12.0) and 47% increase in CS (10.4 ± 2.2) activity at CT_max_, as seen in cytosolic enzymes (Supplementary Table S7). Similarly, RBD had a 20% intraspecific decrease in CS activity at CT_max_ (7.2 ± 1.2) compared with baseline levels (9.0 ± 2.0; Supplementary Table S7). COX CT_max_ activity level had no differences between species, nor any differences within species between treatment groups.

**Figure 4:**
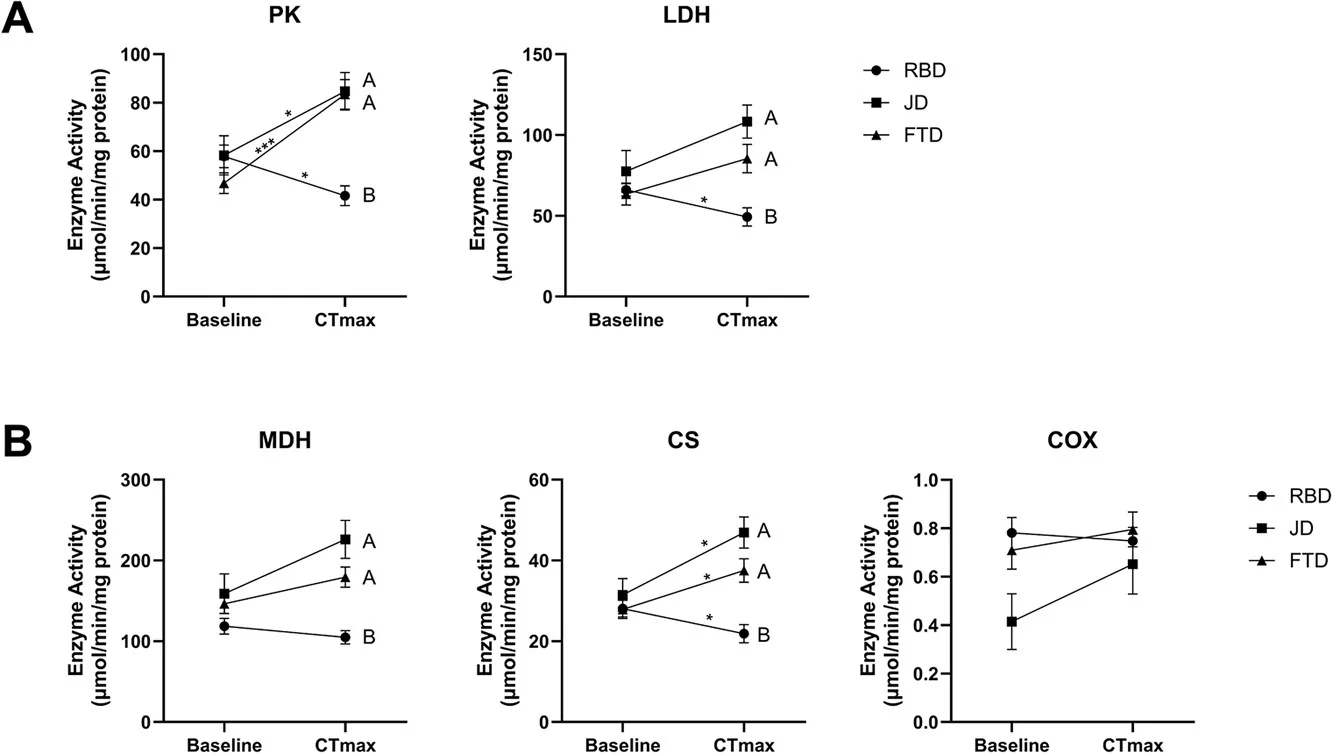
Normalized enzyme activity of RBD, JD and FTD heart tissue at temperature baseline (15°C) and at CT_max_ in cytosolic (A) and mitochondrial (B) enzymes. Interspecies differences in activity at baseline denoted by different lowercase letters, interspecies differences in activity at CT_max_ denoted by different uppercase letters. Intraspecies differences between Baseline and CT_max_ denoted by stars (*P* ≤ 0.05 = *, *P* ≤ 0.01 = **, *P* ≤ 0.001 = ***, *P* ≤ 0.0001 = ****). All differences were determined by a two-way ANOVA with Tukey HSD test (*P* < 0.05; *n* = 10) presented as mean ± SEM. When interaction was significant, data were split by variable and reassessed via one-way ANOVA for interspecific differences, or *t*-test for intraspecific differences. COX data were log transformed to achieve normality.

Within heart tissue, all enzymes had a significant species effect (Fig. 4A and B; Supplementary Table S8). LDH and COX did not have a significant heat treatment effect, and MDH and COX did not express any interactive effect of the two variables (Supplementary Table S8). Heart enzymatic activity across all enzymes displayed no differences between species at baseline temperature levels (Supplementary Tables S9 and S10). However, there was a consistent trend, excluding COX, wherein FTD and JD had higher enzymatic activity than RBD at CT_max_. Intraspecifically, unlike brain tissue, all species had differences between their baseline and CT_max_ enzyme activity levels (Supplementary Table S11). JD had elevated activity at CT_max_ compared to baseline across all enzymes, as observed in brain samples, although this was only significantly different (+45%) in PK (baseline: 58.3 ± 25.6, CT_max_: 84.7 ± 24.6) and (+39%) in CS (baseline: 31.7 ± 3.7, CT_max_: 46.9 ± 12.2). FTD similarly expressed intraspecific increases in enzymatic activity, having 78% higher PK (baseline: 46.8 ± 13.4, CT_max_: 83.5 ± 19.2) and 24% higher CS activity at CT_max_ (37.5 ± 9.2) relative to baseline (27.9 ± 7.2), while RBD activity levels decreased 28% in PK (baseline: 57.9 ± 14.8, CT_max_: 41.6 ± 13.0), 25% in LDH (baseline: 66.1 ± 12.4, CT_max_: 49.3 ± 18.1) and 22% in CS (baseline: 28.1 ± 6.5, CT_max_: 21.9 ± 7.1) at CT_max_. COX showed no differences, neither species- nor treatment-related.

### Respirometry

**Figure 5:**
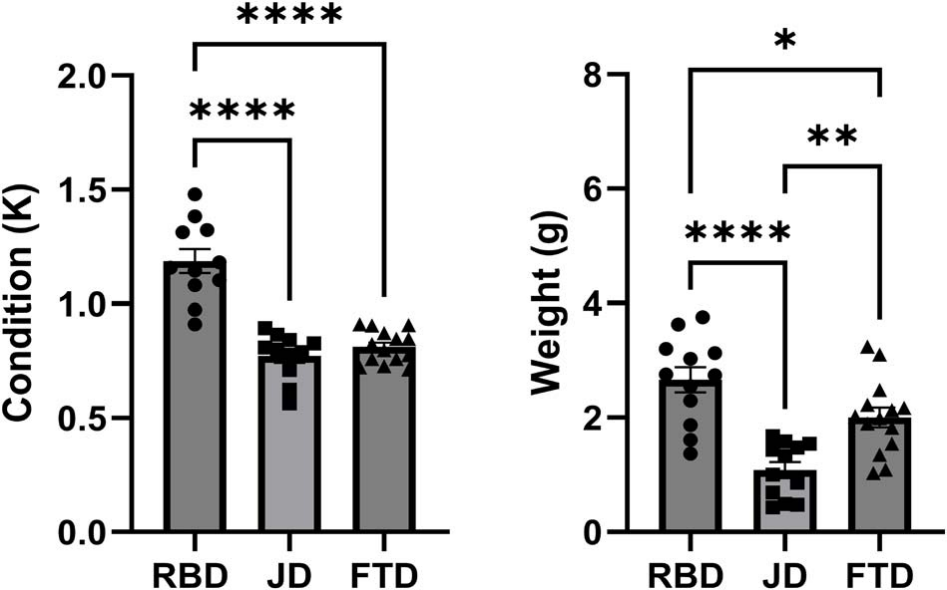
RBD, JD and FTD body size metrics for fish used in respirometry trials. Differences were determined via one-way ANOVA (*P* < 0.05, *n* = 11–14). Dots represent individual data points. Alt Text: Two graphs depicting the relative condition factor and weight differences between Rainbow, Johnny and Fantail darters.

Body mass and condition factor (K) differed significantly between species (one-way ANOVA; F_2,35_ = 18.40, *P* < 0.0001; F_2,33_ = 41.48, *P* < 0.0001; Fig. 5). RBD had the highest condition (1.19 ± 0.17) compared to JD (0.77 ± 0.10) and FTD (0.81 ± 0.07; Tukey HSD, *P* < 0.0001 for both), which were not different from each other, even though JD had a significantly lower mass than FTD (Tukey HSD, *P* = 0.0023). RBD had the largest mass (2.76 ± 0.75 g), followed by FTD (2.00 ± 0.65 g) and JD last (1.077 ± 0.46 g; Tukey HSD, RBD vs JD, *P* < 0.0001; RBD vs FTD, *P* = 0.035; JD vs FTD, *P* = 0.0023). Additionally, no species–mass interaction existed, displaying no differences in mass scaling between species (ANCOVA, F_2,34_ = 0.77, *P* = 0.470).

**Figure 6:**
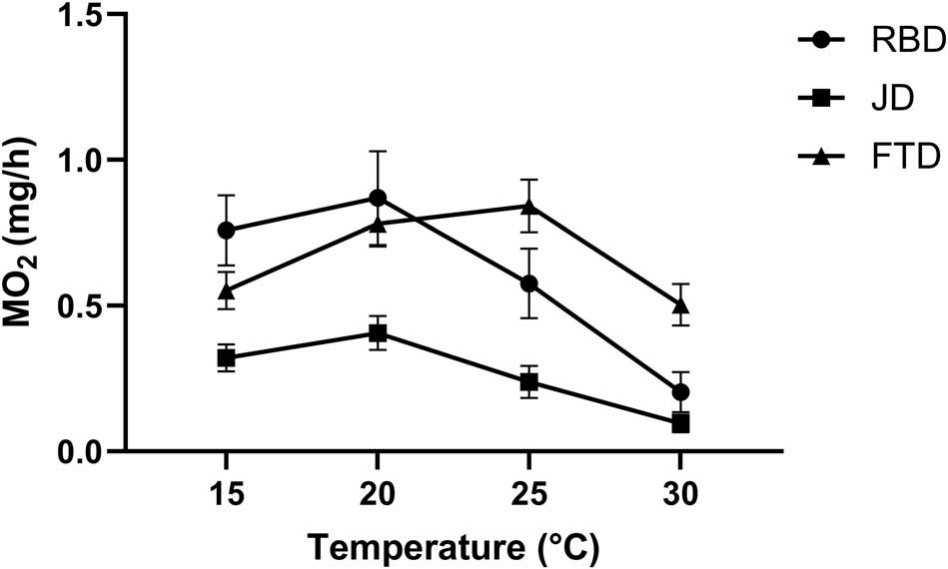
RBD, JD and FTD absolute AS across 15, 20, 25 and 30°C. AS Mass Covariate data accommodated for the impact of mass on MO_2_ and was analysed via mixed effects model and Tukey HSD test (*P* < 0.05; *n* = 14). Individual points represent mean ± SEM. Statistical differences can be found in Tables 1, 2 & 3.

AS differences were significant for all variables; temperature, species and their interaction (Table 1). In AS data split by temperature variable and assessed via linear mixed effects model, both 15 and 20°C had no differences in AS among species (Fig. 6; Table 2). Alternatively at 25°C, FTD had the highest AS (0.84 ± 0.33 mg/h) compared to JD (0.24 ± 0.20; Tukey HSD, *P* = 0.0243) and RBD (0.58 ± 0.41; Tukey HSD, *P* = 0.0008) as well as being higher (0.50 ± 0.23) than JD (0.10 ± 0.10; Tukey HSD, *P* = 0.0009) and RBD (0.20 ± 0.23; Tukey HSD, *P* = 0.0071) at 30°C as well. FTD had a 252% higher AS than JD, and 46% higher than RBD at 25°C, and a 425% and 148% higher AS than JD and RBD at 30°C.

**Table 1:** Linear mixed effects model statistical results on RBD (*n* = 14), JD (*n* = 14) and FTD (*n* = 14) absolute AS measured across 15, 20, 25 and 30°C.

	F	*df*	*P*
Temperature	19.76	3106	**<0.0001**
Species	8.76	2.36	**0.0008**
Interaction	4.067	6106	**0.001**

The model measured the effects of species, temperature and interaction of the two, with mass factored in as a covariate. P-values listed in bold indicate significance.

Alt Text: Table illustrating the statistical results of a linear mixed effects model on AS data. All effects were significant.

Within data split by species (Table 3), RBD and JD had their highest AS values at 20°C (RBD: 0.87 ± 0.56, JD: 0.41 ± 0.22), with RBD decreasing by 76% between 20 and 30°C (30°C: 0.20 ± 0.23; Tukey HSD, *P* = 0.0011), JD by 41% between 20 and 25°C (25°C: 0.24 ± 0.20; Tukey HSD, *P* = 0.0111) and 76% from 20 and 30°C (30°C: 0.10 ± 0.10; Tukey HSD, *P* < 0.0001). FTD AS peaked 5°C higher than JD and RBD, having no differences between 20°C (0.78 ± 0.29) and 25°C (0.84 ± 0.34; Tukey HSD, *P* = 0.8309) but a 35% decrease between 20°C and 30°C (30°C: 0.50 ± 0.25; Tukey HSD, *P* = 0.0004) and a 40% decrease from 25 to 30°C (Tukey HSD, *P* = 0.0004). Mass-corrected data analysed via mixed effects model produced similar results as the mass covariate analysed data, simply with more defined differences with species, reinforcing the overall trends observed (Supplementary Tables S12, S13 and S14).

**Table 2:** Linear mixed effects model with Tukey HSD test results on RBD (*n* = 14), JD (*n* = 14) and FTD (*n* = 14) absolute AS measured across 15, 20, 25 and 30**°**C.

Temperature	Comparison	*P*
15°C	RBD vs JD	0.4551
	RBD vs FTD	0.7546
	JD vs FTD	0.6242
20°C	RBD vs JD	0.2835
	RBD vs FTD	0.1854
	JD vs FTD	0.9670
25°C	RBD vs JD	0.6817
	RBD vs FTD	**0.0008**
	JD vs FTD	**0.0243**
30°C	RBD vs JD	0.735
	RBD vs FTD	**0.0071**
	JD vs FTD	**0.0009**

These data were split by temperature and differences analysed between species within each temperature point. P values listed in bold indicate significance.

Alt Text: Table illustrating Tukey *post hoc* test statistics from the linear mixed effects model on AS data, comparing differences between species within each temperature point.

**Table 3:** Linear mixed effects model with Tukey HSD test results on RBD (*n* = 14), JD (*n* = 14) and FTD (*n* = 14) absolute AS measured across 15, 20, 25 and 30**°**C.

Species	Comparison (°C)	*P*
RBD	15 vs 20	0.7909
	15 vs 25	0.7850
	15 vs 30	**0.0152**
	20 vs 25	0.2571
	20 vs 30	**0.0011**
	25 vs 30	0.1272
JD	15 vs 20	0.3816
	15 vs 25	0.3529
	15 vs 30	**0.0008**
	20 vs 25	**0.0111**
	20 vs 30	**<0.0001**
	25 vs 30	0.0821
FTD	15 vs 20	**0.0135**
	15 vs 25	**0.0011**
	15 vs 30	0.9611
	20 vs 25	0.8309
	20 vs 30	**0.0048**
	25 vs 30	**0.0004**

These data were split by species and differences analysed between temperatures within species.

Alt Text: Table illustrating Tukey *post hoc* test statistics from the linear mixed effects model on AS data, comparing differences within species across all temperatures measured. P values listed in bold indicate significance.

## Discussion

Significant differences in thermal tolerance limits between darter species were observed, with FTD demonstrating the highest CT_max,_ JD a moderate CT_max_ and RBD the lowest. These results align with trends observed by Ingersoll and Claussen (1984), who found FTD to have higher thermal tolerance limits than JD, and with Borowiec *et al*. (2024), who observed nearly identical RBD CT_max_ values as those demonstrated here. It is plausible these differences in darter thermal tolerance limits may be a reflection of species’ previous acclimatization or adaptation to habitat temperature conditions. Each of the three darter species, although closely related, have a distinct microhabitat and, due to their benthic nature, have been recorded to demonstrate high site fidelity, each remaining within its distinctive microhabitat throughout the majority of their lifetime (Hicks and Servos, 2017). FTD, preferring shallow fast-flowing riffles, likely experience the most thermal variability (Kuehne and Barbour, 1983; Dallas and Rivers-Moore, 2011). RBD, residing in deep waters with fast flow, occupy the most thermally stable habitats, and JD, which select stagnant pools and shorelines, inhabit environments likely with intermediate temperature variability (Pratt and Lauer, 2013). Lower thermal tolerances are associated with species from more thermally stable habitats (Farless and Brewer, 2017,), and similar trends have been reported in Orangethroat darters (*Etheostoma spectabile*), where populations from fluctuating thermal environments tended to have a higher CT_max_ compared to those from more fixed conditions (Feminella and Matthews, 1984). Furthermore, Ingersoll and Claussen (1984) attributed their recorded differences between JD and FTD thermal tolerance limits as a result of habitat temperature fluctuation differences (Ingersoll and Claussen, 1984), collectively suggesting that prolonged exposure to thermally variable habitats may be involved in determining RBD, JD and FTD responses to thermal stress. However, it is similarly possible the observed CT_max_ trends could be attributed to microhabitat temperature alone, rather than variability, as it is well established that warm-acclimated fishes have higher thermal tolerances (Comte and Olden, 2017,), and prior research has reported a significant effect of acclimation temperature on CT_max_ in Fantail and Rainbow darters (Hlohowskyj and Wissing, 1985). Here, the depths of RBD, JD and FTD microhabitats would be expected to result in temperatures of low to high, respectively, coinciding with their recorded CT_max_ values, potentially alluding to the influence of acclimatization to different habitat temperatures. Unfortunately, the role of microhabitat thermal conditions in determining thermal tolerance limits was not conclusively determined here, as we did not have definitive results on differences in temperature conditions between RBD, JD and FTD habitats. Regardless, it is still expected that the three darter species experience different thermal profiles, based on historical reports of RBD, JD and FTD habitats, and previous research done on their respective microhabitat temperatures, all of which claim habitat temperature and variability differences (Becker, 1983; Kuehne and Barbour, 1983; Ingersoll and Claussen, 1984; Pratt and Lauer, 2013). Ultimately, further study investigating the influence of habitat temperature, depth and variability on thermal tolerance limits of darters is needed.

RBD, JD and FTD enzyme activity levels at baseline (15°C) and CT_max_ reflected the trends observed in their thermal tolerance limits, with FTD demonstrating elevated enzymatic activity, and RBD low or sometimes decreasing activity. These findings suggest that RBD may not be capable of responding or adjusting to acute temperature exposure, as their lower enzyme activity did not increase, and occasionally decreased, with temperature elevations, potentially hindering the metabolic increases needed to maintain energy demand. Conversely, the higher enzymatic activity in FTD at both baseline and CT_max_ may provide an advantage at handling acute temperature increases, as they are already primed to respond to increased metabolic demands, translating to high thermal tolerance limits. Interestingly, it does appear that enzymatic activity rates observed within each species seem to positively correlate or play a role in their respective thermal tolerance limits, with high enzymatic activities aligning with high CT_max_ values, and low activities with low thermal tolerances. This may be corroborated by previous work comparing the aerobic enzymatic activity levels of white-blooded (hemoglobinless) *Chaenocephalus aceratus* and red-blooded *Notothenia coriiceps*, measured at ambient baseline temperatures and at CT_max_. *Notothenia coriiceps,* which had the higher CT_max_, exhibited greater CS and COX activities, as well as higher ATP levels (O’Brien *et al*., 2018). While white-blooded fishes generally have lower thermal tolerance limits compared to red-blooded, this is not a result of differences in oxygen-carrying capacity (Devor *et al*., 2016). Rather, this work illustrated cardiac mitochondrial function, particularly through increases in aerobic enzymes, may contribute to elevated thermal tolerance limits, suggesting that for both the present study, and the study mentioned, higher enzymatic activities may be responsible for elevated thermal tolerance limits, particularly through improved metabolic performance (O’Brien *et al*., 2018). Thus, correlations may exist between increases in energetic enzyme activity and elevated thermal tolerances, although this has been examined very little, suggesting a need for further investigation.

It was expected that, when comparing baseline and CT_max_ enzymatic activity levels within individual species, activity should increase with acute heat exposure due to inherent biochemical increases in reaction rates (Fry, 1947; Pichaud *et al*., 2017). This was most consistently observed in JD, occasionally in some enzymes of FTD and never in RBD. It is uncertain why JD appeared to be more plastic than the other two species, capable of increasing their enzymatic activity significantly on such acute time scales; however, this again may be a result of the habitat conditions JD populations experience, as it has been shown that fish from more variable microhabitats can exhibit greater plasticity than those from more stable environments (Morgan *et al*., 2022). JD reside in sandy, stagnant pools and shorelines, often with little riparian cover; these regions of the river could potentially heat up several degrees relatively quickly compared to the shallow, but fast-flowing regions that FTD inhabit (Kuehne and Barbour, 1983; Ingersoll and Claussen, 1984). While FTD microhabitats would be assumed to have more temperature variability due to their shallowness, these habitats may experience fluctuations over longer periods of time compared to JD habitats because of increased water flow. Although differences in timescales of acute temperature increases between JD and FTD microhabitats were not collected here, these trends were reflected in the more moderate FTD intraspecific increases in activity: some enzymes showed significant increases in CT_max_ compared to baseline, but were not as pronounced as those observed in JD. Physiologically, it’s unclear what mechanisms specifically are responsible for the striking increases in enzyme activity between baseline JD and CT_max_ JD, although it could be related to adaptive or acclimatization mechanisms such as increases in isozymes, particularly those that may be more efficient at higher temperatures, or elevations in enzyme co-factor levels, both of which could translate to quick elevations in enzymatic activity (Somero and Hochachka, 1968; Somero, 1975; Shaklee *et al*., 1977). Alternatively, RBD likely do not experience acute temperature increases in their deep and fast-flowing habitats, potentially accounting for their inability to induce alterations in enzyme activity at elevated temperatures. It’s possible that acclimatization/adaptation to colder, more stable conditions results in enzyme denaturation occurring at lower temperatures compared to JD and FTD, contributing to the overall lower enzymatic activity levels in RBD at elevated temperatures (Reynolds and Casterlin, 1980; Fields *et al*., 2015); however, pinpointing the exact physiological reasoning behind this is beyond the scope of the present study.

From an organ-level perspective, both brain and heart enzymatic activities exhibited similar results, with the exception of interspecific differences in baseline activity levels, demonstrating no notable differences between aerobic and anaerobic enzymes measured. This may be due to the reliance of brain and heart tissue almost entirely on aerobic processes (Soengas and Aldegunde, 2002), resulting in relatively minor shifts to anaerobic metabolism that simply were not detected, or due to the short timescale of the CT_max_ trial (~1 h), which may not have provided enough time to allow a full initiation of anaerobic pathways. Elevations in anaerobic activity and decreases in aerobic enzyme activity levels may be more apparent in tissue like muscle, which is more capable of sustaining hypoxic conditions, and thus would clearly depict the shift from aerobic to anaerobic energy production due to low tissue oxygen as a result of acute temperature increases.

The intra- and interspecific differences in AS among darters, especially the elevated rates observed in FTD, align with the patterns observed in the CT_max_ and enzyme activity experiments. FTD’s superior AS and their ability to sustain it at higher temperatures before metabolic failure, correspond to their high CT_max_ and enzymatic activities, supporting their greater metabolic capacity and thermal tolerance. The observed differences in AS may again be a reflection of FTD’s warm but thermally variable habitat conditions. Previous studies have found fish from variable conditions to exhibit higher and broader AS across larger temperature ranges compared to those kept in stable conditions in a lab, further supporting the potential influence of microhabitat on resilience to temperature increases (Morgan *et al*., 2022). Additionally, FTD mobility may also play a significant role in their high AS. More mobile species, observed in fishes from faster flowing environments, are known to have an elevated AS, potentially due to their ability to maintain higher levels of energy expenditure (Peck *et al*., 2009; Fu *et al*., 2022).

Like FTD, RBD’s metabolic responses correspond with their expected microhabitat, cold and thermally stable, which may be partly responsible for their lower AS at temperature extremes, considering their adaptation and acclimatization to colder environments may render these species metabolically incapable of responding to temperature elevations compared to other species from warmer habitats. Furthermore, given their reported preference for colder environments (Wichert and Lin, 1996; Coker *et al*., 2001), exposure temperatures used in the AS trials may have exceeded the optimal range at which RBD achieve their highest AS.

Conversely, the low AS of JD was surprising, as it was assumed that smaller fish from more thermally variable habitats would exhibit high AS, capable of withstanding acute temperature increases. This may indicate that mobility, and/or other physiological or lifestyle differences, may have a greater impact than habitat temperature, especially considering their selection of stagnant waters, notably pools or shorelines (Kuehne and Barbour, 1983; Ingersoll and Claussen, 1984) which would exert minimal energetic influence and suggest that they move very little. Consequently, JD’s limited movement may result in lower metabolic functions, making them incapable of handling increased energetic demands associated with acute temperature elevations.

Albeit, mobility differences between darters were not examined in the present study, and while RBD have been shown to have a minimal mobility of ~5 m (Hicks and Servos, 2017), differences in movement between benthic RBD, JD and FTD species have not been compared in the broader literature. Thus, it remains inconclusive what external influences, whether that be movement or temperature-related, or something else entirely, may be responsible for the interspecific metabolic differences observed.

Additionally, it is worth noting that the experimental design employed in the AS experiment, considering it consisted of acute heat stress followed by a week of rest, may have inadvertently resulted in a ‘heat hardening’ response within RBD, JD and FTD (Maness and Hutchison, 1980). Since the same fish underwent four consecutive temperature ramping trials, some acclimation may have been induced as they progressed through the trials, potentially resulting in an over-exaggerated resilience to the temperatures stressors introduced later on in the study (Schaefer and Ryan, 2006; Farless and Brewer, 2017). It is therefore possible that RBD JD and FTD AS is actually lower at temperature extremes than observed.

While thermal tolerance limits, enzyme activity and AS of each species appear to align well with what would be expected of the temperature conditions and variability of their respective microhabitats, it is likely that habitat acclimatization and/or adaptation is not solely responsible for the interspecific differences observed between RBD, JD and FTD. Life history, activity and environmental conditions like temperature and oxygen level can greatly impact metabolism and thus physiological responses to environmental temperature increases in fish, along with differences in the evolutionary history of species (Peck *et al*., 2009; Comte and Olden, 2017; Norin and Speers-Roesch, 2021; Fu *et al*., 2022) Regardless, FTD may be the best equipped at responding to temperature-driven increased metabolic demands given their elevated baseline enzymatic activity and broader AS, with this advantage supported by their superior thermal tolerance limits. In contrast, RBD are likely the most vulnerable to acute heat stress due to their low CT_max,_ and reduced energetic enzymatic activity and AS at elevated temperatures. Although JD exhibited a similarly low AS as RBD, their elevations in enzyme activity at temperature extremes and higher thermal tolerance limits suggest that they may be more resilient than RBD, but less so than FTD. Given that the darters' responses in the acute CTmax trial, which involved an approximate 1-hour temperature ramp from 15°C to ~30°C, closely mirrored the general trends seen in the AS heating profiles, it is possible that the relative thermal tolerance limits observed for RBD, JD, and FTD in this study reflect their ability to cope with acute heatwaves in their natural environments.

Irrespective of their particular interspecific differences, all species’ AS began declining significantly at temperatures well before their thermal tolerance limits, suggesting that even at temperatures as low as 20–25°C, RBD, JD and FTD may experience metabolic challenges and may have to allocate more energy towards accommodating these temperature increases. Additionally, while it is known that acclimatization and/or adaptation to warmer habitats may translate to a higher CT_max_ in fish, this often comes at the cost of a lower thermal safety margin, the difference between acclimation temperature and their thermal tolerance limits, making these species still vulnerable to further environmental warming (Campos *et al*., 2018). In other words, while the results here do point to an FTD advantage, their protection against elevated water temperatures is not a guarantee; rather, if average temperatures and extreme heatwaves continue, all of these darters will likely be negatively impacted.

As of July 2024, live, publicly available water temperature monitoring of the Grand River, home to the darters used in this study, logged river temperatures as high as 29.2°C (Grand River Conservation Authority, 2022). These temperatures surpass the levels demonstrated here to induce aerobic decline, and are dangerously close to the thermal tolerance limits of RBD, JD and FTD, indicating that these species are already at a serious risk of being adversely impacted, and may be vulnerable to extirpation if extreme heat events continue to rise. Given that similar warming trends and increased temperature variability have been observed and projected for much of darters’ North American range (Deen *et al*., 2021), other populations will likely face similar challenges. RBD, JD and FTD will therefore likely need to make metabolic trade-offs to maintain physiological homeostasis, and consequently, less energy may be available for processes like growth and reproduction, potentially resulting in decreases in population sizes.

Globally, the magnitude of mass mortality events have been increasing in intensity for fishes since 1940 (Fey *et al*., 2015). Elucidating the metabolic, and overall temperature-dependent, responses of small, freshwater fishes to elevated temperatures is therefore of paramount importance for protecting riverine ecosystems. Employment of the approaches used here, specifically the combination of short- and long-term heat exposures in tandem with biochemical and whole-body analysis, can be applied to at-risk species and others, to provide understanding of how these fishes, and the broader freshwater ecosystems that rely on them, may be impacted by climate change. These findings can inform conservation initiatives, such as restoring riparian vegetation to mitigate temperature fluctuations or conducting population habitat relocations (Cooke and Suski, 2008), which may help to protect and support fish populations amidst a warming climate.

## Supplementary Material

Web_Material_coaf027

## Data Availability

The data presented in this article will be shared on reasonable request to the corresponding author.
