# Correction to: Characterization of darter (Etheostoma spp.) interspecific energetic responses to acute temperature elevations

**DOI:** 10.1093/conphys/coag012

**Published:** 2026-02-14

This is a correction to: Allison V Weber, Paul M Craig, Characterization of darter (Etheostoma spp.) interspecific energetic responses to acute temperature elevations, *Conservation Physiology*, Volume 13, Issue 1, 2025, coaf027, 10.1093/conphys/coaf027.

 In the originally published version of this manuscript, the following statement was omitted from the Funding section:

 ``This project was additionally funded through a Fisheries and Oceans Canada (DFO) Canada Nature Fund for Aquatic Species at Risk Grant (Agreement Number: 2023-NF-OPR-025).''

 This has been corrected and the Funding section now reads as follows:

 This work was supported and funded through a Natural Science and Engineering Research Council (NSERC) Discovery Grant (RGPIN-2022-03202) to P.M.C. Infrastructure used in this study was funded through an Innovation Fund 2020 to P.M.C. from the Canadian Foundation for Innovation (39560). This project was additionally funded through a Fisheries and Oceans Canada (DFO) Canada Nature Fund for Aquatic Species at Risk Grant (Agreement Number: 2023-NF-OPR-025). 

