# Supplementary material for: Characterization of darter (Etheostoma spp.) interspecific energetic responses to acute temperature elevations
Source: Conserv Physiol. 2025 Apr 15;13(1):coaf027. doi: 10.1093/conphys/coaf027 (PMC11998911; doi:10.1093/conphys/coaf027)
Supplement: Web_Material_coaf027 [file web_material_coaf027.zip › Supplementary.pdf]

Supplementary:

Table S1: Body mass of Rainbow (RBD, n=14), Johnny (JD, n=14) and Fantail (FTD, n=14) used in respirometry experimentation was ln transformed and plotted against aerobic scope (mg/h) measured at 15°C to assess for linear model fit, with the following R^2 recorded.

Species	R^2
RBD	0.68
JD	0.65
FTD	0.57

Table S2: Repeated measures Two Way ANCOVA to assess the impact of mass on respirometry data analysis. Weight proved to be a significant covariate.

Metric	F	df	p
Weight	50.5788	1,38	<0.0001

Table S3: ANCOVA performed on CT_{max} and body mass to assess the impact of mass on thermal tolerance limits.

Metric	F	df	p
Weight	2.3533	2,54	0.1047

Table S4: Brain enzyme activity Two-Way ANOVA statistical results for pyruvate kinase (PK), lactate dehydrogenase (LDH), malate dehydrogenase (MDH), citrate synthase (CS), and cytochrome c oxidase (COX). n=10 for each enzyme.

Enzyme	Variable	F	df	p
PK	Interaction	9.115	2,54	0.0004
	Species	54.91	2,54	<0.0001
	CT_{max}	0.0595	1,54	0.8082
LDH	Interaction	13.35	2,54	<0.0001
	Species	12.23	2,54	<0.0001
	CT_{max}	1.275	1,54	0.2639
MDH	Interaction	10.79	2,54	0.0001
	Species	49.42	2,54	<0.0001
	CT_{max}	5.728	1,54	0.0202
CS	Interaction	11.05	2,54	<0.001
	Species	15.75	2,54	<0.001
	CT_{max}	0.324	1,54	0.5716
COX	Interaction	2.943	2,54	0.0612
	Species	9.874	2,54	0.0002
	CT_{max}	3.305	1,54	0.0746

Table S5: Brain enzyme activity One-Way ANOVA statistical results. This was data used above (S4) in the initial Two-Way ANOVA and then split by heat treatment to examine for differences between species at baseline and at CTmax treatments. Tukey post hoc tests on the One-Way ANOVA can be found in Table S6. Since the interaction term of the Two-Way ANOVA was not significant for COX, that data was not split by variable and thus not included in this analysis. n=10 for all enzymes.

Enzyme		F	df	p
PK	Baseline	29.27	2,27	<0.0001
	CTmax	39.01	2,27	<0.0001
LDH	Baseline	13.78	2,27	<0.0001
	CTmax	9.456	2,27	0.0008
MDH	Baseline	18.49	2,27	<0.0001
	CTmax	74.00	2,27	<0.0001
CS	Baseline	11.48	2,27	0.0002
	CTmax	17.56	2,27	<0.0001

Table S6: Brain enzyme activity of Johnny Darter (JD, n = 10), Rainbow Darter (RBD, n = 10), and Fantail Darter (FTD, n = 10) at baseline temperatures of 15°C and at Critical Thermal Maximum (CT_{max}), compared between species using a One-Way ANOVA and Tukey's Honest Significant Difference (HSD) test. Statistical differences are visualized in Figure 3. Enzymes measured included pyruvate kinase (PK), lactate dehydrogenase (LDH), malate dehydrogenase (MDH), and citrate synthase (CS). Because the interaction term of the Two-Way ANOVA was not significant for cytochrome c oxidase (COX), that data was not split by variable and thus not included in this analysis.

Enzyme & Treatment	Comparison	p
PK Baseline	RBD vs JD	**0.0147**
	RBD vs FTD	**0.0003**
	JD vs FTD	**<0.0001**
PK CT_{max}	RBD vs JD	**0.0301**
	RBD vs FTD	**<0.0001**
	JD vs FTD	**<0.0001**
LDH Baseline	RBD vs JD	**0.0142**
	RBD vs FTD	0.0906
	JD vs FTD	**<0.0001**
LDH CT_{max}	RBD vs JD	**0.0027**
	RBD vs FTD	**0.002**
	JD vs FTD	0.992
MDH Baseline	RBD vs JD	0.8245
	RBD vs FTD	**<0.0001**
	JD vs FTD	**0.0001**
MDH CT_{max}	RBD vs JD	**<0.0001**
	RBD vs FTD	**<0.0001**
	JD vs FTD	0.6331
CS Baseline	RBD vs JD	0.0991
	RBD vs FTD	**0.0354**
	JD vs FTD	**0.0002**
CS CT_{max}	RBD vs JD	**0.0002**
	RBD vs FTD	**<0.0001**
	JD vs FTD	0.7595

Table S7: T-test statistical results for intraspecific differences in brain enzyme activity of Rainbow Darter (RBD, n =10), Johnny Darter (JD, n=10), and Fantail Darter (FTD, n=10), across heat treatments. Statistical differences visualized in Figure 3. Enzymes measured included pyruvate kinase (PK), lactate dehydrogenase (LDH), malate dehydrogenase (MDH), and citrate synthase (CS). Because the interaction term of the Two-Way ANOVA was not significant for cytochrome c oxidase (COX), that data was not split by variable and thus not included in this analysis.

Endpoint	t	df	p
RBD PK	2.240	18	**0.0379**
RBD LDH	1.550	18	0.1385
RBD CS	2.469	18	**0.0238**
RBD MDH	0.6149	18	0.5463
JD PK	3.087	18	**0.0064**
JD LDH	4.258	18	**0.0005**
JD CS	3.659	18	**0.0018**
JD MDH	5.242	18	**<0.0001**
FTD PK	1.216	18	0.2396
FTD LDH	1.641	18	0.1181
FTD CS	1.006	18	0.3276
FTD MDH	0.4213	18	0.6785

Table S8: Heart enzyme activity Two-Way ANOVA statistical results for pyruvate kinase (PK), lactate dehydrogenase (LDH), malate dehydrogenase (MDH), citrate synthase (CS), and cytochrome c oxidase (COX). n=10 for each enzyme.

Enzyme		F	df	p
PK	Interaction	10.78	2,54	0.0001
	Species	6.826	2,54	0.0023
	CT_{max}	9.989	1,54	0.0026
LDH	Interaction	4.33	2,54	0.0180
	Species	8.433	2,54	0.0006
	CT_{max}	2.917	1,54	0.0934
MDH	Interaction	3.077	2,54	0.0542
	Species	12.36	2,54	<0.0001
	CT_{max}	4,639	1,54	0.0357
CS	Interaction	6.951	2,54	0.0021
	Species	11.18	2,54	<0.0001
	CT_{max}	6.587	1,54	0.0131
COX	Interaction	1.171	2,54	0.3177
	Species	4.321	2,54	0.0182
	CT_{max}	1.784	1,54	0.1872

Table S9: Heart enzyme activity One-Way ANOVA statistical results. This was data used above in the initial Two-Way ANOVA and then split by heat treatment to examine for differences between species at baseline and at CTmax treatments. Since the interaction term of the Two-Way ANOVA was not significant for COX or MDH, that data was not split by variable and thus not included in this analysis.

Enzyme		hF	df	p
PK	Baseline	1.220	2,27	0.3109
	CTmax	15.76	2,27	<0.0001
LDH	Baseline	0.7465	2,27	0.4826
	CTmax	12.37	2,27	0.0002
CS	Baseline	0.4737	2,27	0.6278
	CTmax	16.84	2,27	<0.0001

Table S10: Heart enzyme activity of Johnny Darter (JD, n = 10), Rainbow Darter (RBD, n = 10), and Fantail Darter (FTD, n = 10) at baseline temperatures of 15°C and at Critical Thermal Maximum (CT_{max}), compared between species using a One-Way ANOVA and Tukey's Honest Significant Difference (HSD) test. Statistical differences are visualized in Figure 4. Enzymes measured included pyruvate kinase (PK), lactate dehydrogenase (LDH), and citrate synthase (CS). Because the interaction term of the Two-Way ANOVA was not significant for cytochrome c oxidase (COX), or malate dehydrogenase (MDH) that data was not split by variable and thus not included in this analysis.

Enzyme & Treatment	Comparison	p
PK Baseline	RBD vs JD	0.9988
	RBD vs FTD	0.3919
	JD vs FTD	0.3674
PK CTmax	RBD vs JD	**0.0001**
	RBD vs FTD	**0.0002**
	JD vs FTD	0.9886
LDH Baseline	RBD vs JD	0.624
	RBD vs FTD	0.9748
	JD vs FTD	0.4929
LDH CTmax	RBD vs JD	**0.0001**
	RBD vs FTD	**0.0147**
	JD vs FTD	0.1539
CS Baseline	RBD vs JD	0.6987
	RBD vs FTD	0.9981
	JD vs FTD	0.6631
CS CTmax	RBD vs JD	**<0.0001**
	RBD vs FTD	**0.0037**
	JD vs FTD	0.0957

Table S11: T-test statistical results for intraspecific differences in heart enzyme activity of Rainbow Darter (RBD, n =10), Johnny Darter (JD, n=10), and Fantail Darter (FTD, n=10), across heat treatments. Statistical differences visualized in Figure 4. Enzymes measured included pyruvate kinase (PK), lactate dehydrogenase (LDH), and citrate synthase (CS). Because the interaction term of the Two-Way ANOVA was not significant for cytochrome c oxidase (COX) or malate dehydrogenase (MDH), that data was not split by variable and thus not included in this analysis.

Endpoint	t	df	p
RBD PK	2.618	18	**0.0174**
RBD LDH	2.793	18	**0.0120**
RBD CS	2.383	18	**0.0284**
JD PK	2.354	18	**0.031**
JD LDH	1.864	18	0.0788
JD CS	2.765	18	**0.0127**
FTD PK	4.950	18	**0.0001**
FTD LDH	1.983	18	0.0628
FTD CS	2.602	18	**0.0180**

Table S12: Linear mixed effects model statistics performed on Mass Corrected $\dot{M}O_2$ (mg O^2 kg^{-1} hr^{-1}) Aerobic Scope data.

	F	df	p
Temperature	25.84	3,103	<0.0001
Species	9.942	2,39	0.0003
Interaction	4.164	6,103	0.0009

Table S13: Linear mixed effects model with Tukey's Honest Significant Difference (HSD) test separated by temperatures on Mass Corrected $\dot{M}O_2$ (mg O^2 kg^{-1} hr^{-1}) Aerobic Scope data. n = 14 per Rainbow darter (RBD), Johnny darter (JD), and Fantail darter (FTD).

Temperature	Comparison	p
15°C	RBD vs JD	0.8258
	RBD vs FTD	0.9876
	JD vs FTD	0.8915
20°C	RBD vs JD	0.4627
	RBD vs FTD	0.2566
	JD vs FTD	0.9113
25°C	RBD vs JD	0.9971
	RBD vs FTD	<0.0001
	JD vs FTD	<0.0001
30°C	RBD vs JD	0.9511
	RBD vs FTD	0.0026
	JD vs FTD	0.0007

Table S14: Linear mixed effects model with Tukey's Honest Significant Difference (HSD) test separated by species on Mass Corrected $\dot{M}O_2$ (mg O^2 kg^{-1} hr^{-1}) Aerobic Scope data. n = 14 per Rainbow darter (RBD), Johnny darter (JD), and Fantail darter (FTD).

Species	Comparison (°C)	p
RBD	15 vs 20	0.608
	15 vs 25	0.6452
	15 vs 30	0.0015
	20 vs 25	0.0775
	20 vs 30	<0.0001
	25 vs 30	0.0444
JD	15 vs 20	0.188
	15 vs 25	0.2005
	15 vs 30	<0.0001
	20 vs 25	0.0008
	20 vs 30	<0.0001
	25 vs 30	0.016
FTD	15 vs 20	0.0199
	15 vs 25	0.0032
	15 vs 30	0.9856
	20 vs 25	0.9345
	20 vs 30	0.0105
	25 vs 30	0.0017
